# Fungi and earthworm abundance and diversity as affected by biochar and mulching amendments in ethiopian mustard production

**DOI:** 10.1371/journal.pone.0343099

**Published:** 2026-03-10

**Authors:** Naza Emanuel Mmbaga, Stanslaus Terengia Materu

**Affiliations:** 1 Department of Biology, College of Natural and Mathematical Sciences, University of Dodoma, Dodoma, Tanzania; 2 Department of Civil and Water Resources Engineering, School of Engineering Sciences and Technology, Sokoine University of Agriculture, Morogoro, Tanzania; ICAR Research Complex for Eastern Region, INDIA

## Abstract

Organic agriculture relies on the in-field generation of nutrients through the decomposition and mineralization of organic matter (OM). Soil macro- and micro-organisms are vital for this self-sustaining nutrient production; however, insufficient organic matter, limited microorganisms, and poor soil conditions can impede the process. This study investigated the effects of biochar and mulching on the abundance and diversity of soil microorganisms (fungi) and macro-organisms (earthworms) under Ethiopian mustard (*Brassica carinata*) cultivation over two growing seasons in 2023. The site featured loamy soil with a pH of 5.5–6.0. Treatments included rice husks biochar (5 t/ha); rice husk mulching and compared to a control, using a completely randomized block design with three replicates. Measurements included fungal colony abundance, earthworm frequency, Simpson diversity index, and soil moisture retention under rainfed conditions with minimal irrigation during dry spells. Results showed that at a 5 cm soil depth, biochar increased fungal abundance by 32.05% compared to mulching, and by 113.35% relative to the control. Mulching also improved colony abundance by 61.57% over the control. At a depth of 10 cm, biochar enhanced colony abundance by 42.14% compared to mulching and by 42.82% relative to the control. The highest diversity index (0.596) was observed in biochar-treated blocks, while the control had the lowest (0.422). Earthworms were the most abundant macro-organisms in both treatments. Biochar’s characteristics may help recondition poor, acidic soils, improving conditions for macro and micro-organisms, thereby enhancing soil health and productivity. These microbial improvements could benefit not only Ethiopian mustard but also major cereal cropping systems. Biochar consistently increased fungal abundance and earthworm frequency across both season.

## 1 Introduction

*Brassica carinata*, or Ethiopian mustard, is gaining popularity as a leaf vegetable and oilseed crop in both rural and urban areas. This species thrives best at mid to high altitudes (2000–2600 m) in fertile, well-drained soils rich in organic matter [1]. Despite its desirable traits, such as heat and drought tolerance, its growth is often limited by unfavorable soil conditions, particularly low pH and nutrient availability [2]. Poor microbial activity in the root zone can further exacerbate challenges related to nutrient mobilization and mineralization [3,4], highlighting the need for improved soil management practices to enhance *Brassica carinata*’s production potential.

Biochar, a carbon-rich soil amendment produced through pyrolysis, has shown promise in improving soil properties [5,6]. It can enhance fertility, porosity, and nutrient retention [7,8], while also serving as a long-term carbon sink [9,10]. However, the adoption of biochar in vegetable production systems remains limited, warranting further exploration of its benefits.

Mulching, another critical soil management practice, involves covering the soil surface to optimize conditions for crop growth [11]. While effective in various contexts, its impact on soil microorganisms and nutrient dynamics in conjunction with biochar is not well understood.

Despite extensive research on biochar and mulching separately, there is a notable gap in comparative studies addressing their combined effects on soil water retention, macro and microorganism abundance, and diversity in the root zone of *Brassica carinata*. This study aims to fill this gap by assessing the contributions of biochar and mulching to soil health and nutrient use efficiency, ultimately providing farmers with insights to enhance production and improve living standards.

## 2 Methodology and materials

### 2.1 Study area

The study was conducted at the Teaching and Research Farms of the College of Natural and Mathematical Sciences (CNMS), University of Dodoma, located in Dodoma City, Tanzania, at latitude 6°10’19” S and longitude 35°44’22” E. The region experiences a semi-arid climate with a long dry season from late April to early December and a short wet season with an average annual rainfall of 570 mm. Land preparation followed conventional farmer practices, involving manual clearing using tools such as hoes, cutlasses, mattocks, and rakes to ensure a uniform seedbed for Ethiopian mustard (*Brassica carinata*) cultivation.

### 2.2 Soil general conditions

The experimental site featured loamy soil with distinct physico-chemical properties in the 0–20 cm surface layer, as detailed in Table 1. The soil had a pH of 5.35, indicating moderate acidity, which can limit nutrient availability. Total nitrogen (TN) content was low at 0.01%, reflecting poor fertility. Phosphorus (P) levels were measured at 1.57 g kg ⁻ ¹, and exchangeable potassium (K) was 60.38 mg kg ⁻ ¹, both suggesting limited nutrient reserves. The cation exchange capacity (CEC) was 20.52 Cmol kg ⁻ ¹, indicating moderate capacity to retain cations. Total organic carbon (TOC) was 4.29 g kg ⁻ ¹, reflecting modest organic matter content. In contrast, the rice straw biochar used had a pH of 10.13, TN of 1.04%, P of 4.29 g kg ⁻ ¹, CEC of 37.84 Cmol kg ⁻ ¹, exchangeable K of 28.42 mg kg ⁻ ¹, TOC of 6.83 g kg ⁻ ¹, specific surface area (SSA) of 79.32 m² g ⁻ ¹, and ash content of 21.92%, indicating its potential to enhance soil fertility and structure.

**Table 1:** Physico-chemical properties of surface soil (0-20 cm) and rice straw biochar.

Parameter	Soil	Biochar
pH	5.35	10.13
TN (%)	0.01	1.04
P (g kg-1)	1.57	4.29
CEC (Cmol kg-1)	20.52	37.84
Exchangeable K (mg kg-1)	60.38	28.42
TOC (g kg-1)	4.29	6.83
SSA (m2 g-1)	–	79.32
Ash (%)	–	21.92

NB: CEC: cation exchange capacity; TOC: total organic carbon; TN: total nitrogen; SSA: specific surface area, K: Potassium and P: Phosphorus

### 2.3 Design and layout

**Fig 1:**
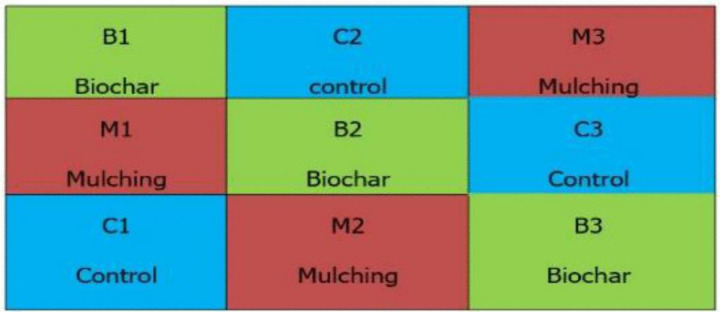
Randomization completely designs for the study.

A completely randomized block design (CRBD) was employed, consisting of three blocks, each containing three plots, totaling nine plots. Each plot measured 3.6 m in width and 4 m in length, covering an area of 14.4 m². Plots within blocks were spaced 50 cm apart, and blocks were separated by 50 cm to minimize interference. Ethiopian mustard seeds were broadcasted uniformly across all plots. Treatments included rice husk biochar, rice husk mulching, and a control, applied as shown in Fig 1. Biochar was incorporated manually into the soil to a depth of 15 cm using a hand hoe to ensure even distribution and integration with the soil matrix.

### 2.4 Sample collection

Soil samples were collected to establish baseline physico-chemical properties before the experiment and during the experiment to monitor changes. Baseline samples were taken from the 0–20 cm soil layer across the experimental site to characterize initial conditions. During the two growing seasons in 2023, soil samples were collected at three random points per plot at depths of 5 cm and 10 cm below the soil surface. These samples were placed in sterile containers and transported to the laboratory for analysis. Earthworms were observed and counted in situ during sampling to assess macro-organism abundance. Fungal populations were quantified using a dilution plating method, where soil samples were diluted in sterile solutions and plated on potato dextrose agar (PDA) media. Fungal colonies were counted, and their morphology was examined under a microscope for species identification. The Simpson diversity index (Equation 1) was calculated to assess fungal diversity, where D ranges from 0 (no diversity) to 1 (infinite diversity), with n as the number of organisms of a particular species and N as the total number of organisms across all species.


$$\text{D}=\text{1}-(\frac{\sum \text{n}(\text{n}-\text{1})}{\text{N}(\text{N}-\text{1})})$$ 1

Where, value of D ranges between 0 and 1. With this index, 1 represents infinite diversity and 0, no diversity, n = the total number of organisms of a particular species and N = the total number of organisms of all species. The Simpson diversity index ranges from 0 to 1, where 0 represents no diversity and 1 represents infinite diversity.

### 2.5 Biochar and mulching application rates

Biochar was applied at a rate of 5 t/ha, based on recommendations from Materu et al. [12], which demonstrated enhanced nutrient retention, increase pH and microbial activity at this rate in similar soil conditions. Rice husk mulch was applied at 5 cm depth and 10 cm depth, aligned with local agricultural practices in Dodoma to optimize soil moisture retention, and suppress weeds. These rates were selected to balance efficacy and practicality, considering the availability of rice husks in the region and their proven benefits in improving soil health [12]. Biochar was incorporated into the soil to a depth of 15 cm, while mulch was spread evenly over the soil surface to maximize its protective and moisture-retaining effects.

### 2.6 Plot management

Plots were managed over two cropping seasons in 2023 under rainfed conditions, with minimal supplementary irrigation applied during dry spells to ensure crop survival. Irrigation was scheduled based on soil moisture monitoring, using a tensiometer to maintain adequate moisture levels without waterlogging. Weeding was performed manually every two weeks to control weed competition, ensuring that Ethiopian mustard growth was not impeded. Pest and disease control followed integrated pest management (IPM) principles, relying on physical and biological methods, such as hand-picking pests and introducing natural predators, to avoid chemical inputs and maintain organic conditions.

### 2.7 Fertilizer use

No additional fertilizers were applied to the plots to isolate the effects of biochar and mulching on soil microbial activity and nutrient dynamics. This approach ensured that observed changes in fungal and earthworm abundance, as well as soil properties, were attributable solely to the biochar and mulching treatments, providing a clear assessment of their efficacy in enhancing soil health under organic farming conditions.

### 2.8 Agronomic practices

Weed management was conducted through manual weeding every two weeks, using hand hoes to remove weeds without disturbing the soil structure or the applied treatments. This method was chosen to align with local organic farming practices and to minimize soil disruption. Pest and disease control adhered to integrated pest management (IPM) strategies, avoiding chemical pesticides to maintain the organic integrity of the experiment. Techniques included regular monitoring, manual removal of pests, and encouraging natural predators such as ladybugs for aphid control. These practices ensured that the study reflected sustainable agricultural methods suitable for smallholder farmers in the region.

### 2.9 Statistical analysis

Data were analyzed using the agricolae package [13] in R software (version 4.2.1). Analysis of variance (ANOVA) was performed with replication as the random effect to assess the significance of treatment effects on fungal abundance, earthworm frequency, and diversity indices. Tukey’s Honestly Significant Difference (HSD) tests were used to compare treatment means at a 0.05 significance level, ensuring robust statistical comparisons across the biochar, mulching, and control treatments.

## 3 Results

### 3.1 Fungi colony abundance

**Fig 2:**
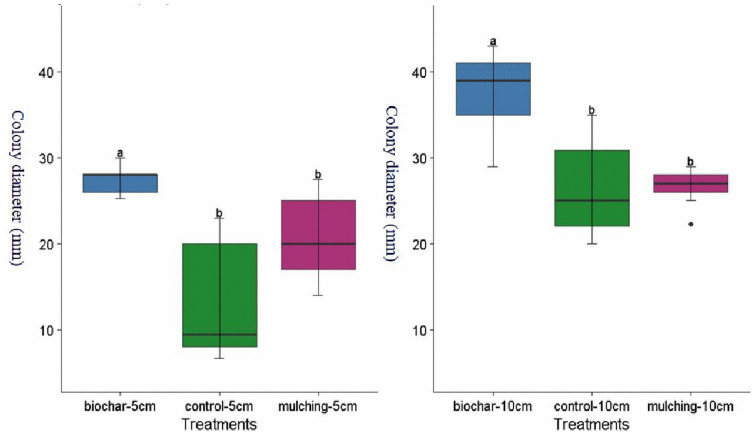
Effect of Biochar and mulching at different soil depth 5 cm (a) and 10 cm (b) on colony size. Error bars represent standard errors of the mean (SEM) based on three replicates per treatment.

**Fig 3:**
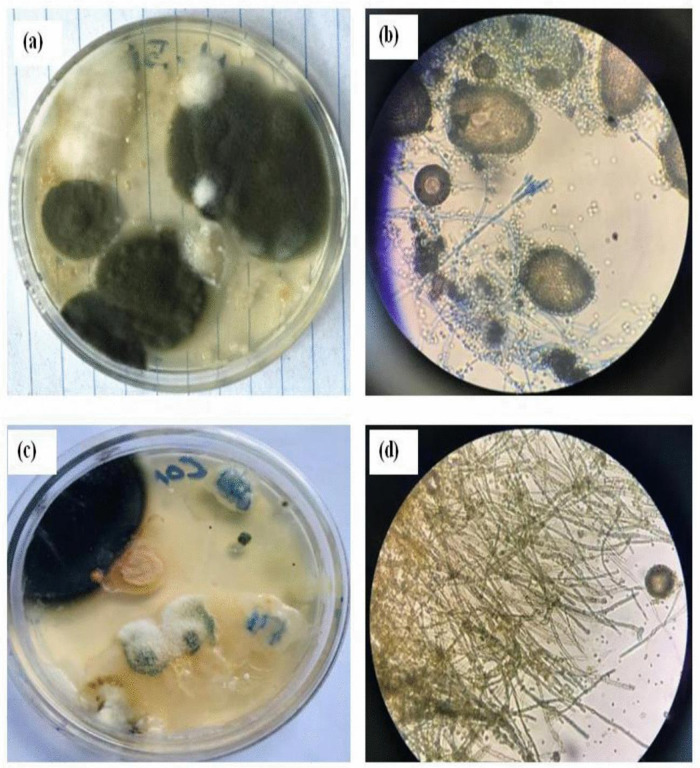
Control plotes: Aspergillus and Penicillin at 5 cm soil depth, plate sample (a) and microscopic photo (b); Aspergillus at 10 cm soil depth, plate sample (c) and microscopic photo (d).

**Fig 4:**
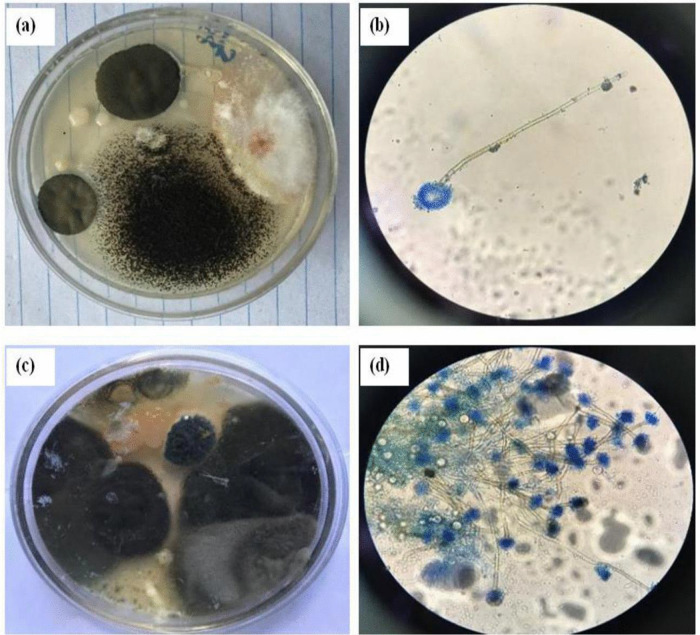
Biochar plots: Aspergillus and Penicillin at 5 cm soil depth, plate sample (a) and microscopic photo (b); Aspergillus and Penicillin at 10 cm soil depth, plate sample (c) and microscopic photo (d).

**Fig 5:**
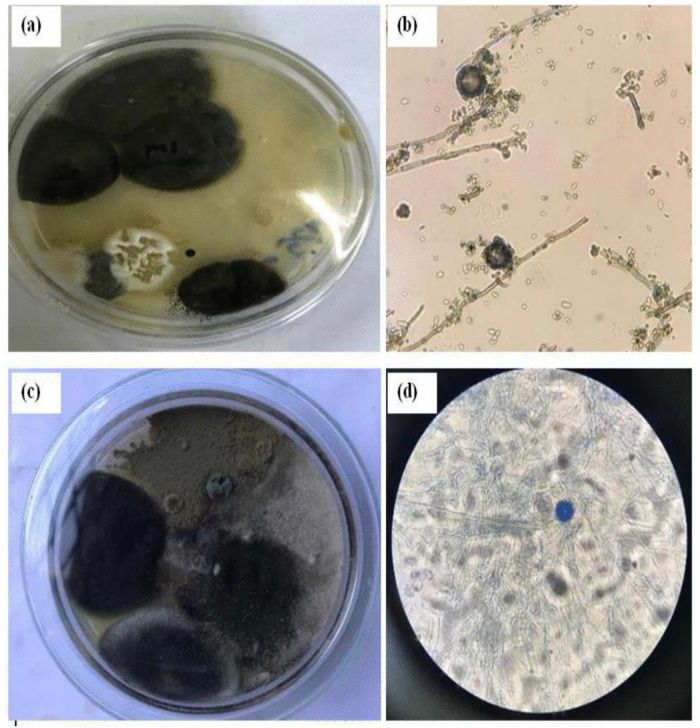
Mulching plots: Aspergillus and Penicillin at 5 cm soil depth, plate sample (a) and microscopic photo (b); Aspergillus at 10 cm soil depth, plate sample (c) and microscopic photo (d).

Colony abundance was significantly influenced (P < 0.05) by biochar and mulching at different soil depths (Fig 2). At a depth of 5 cm, biochar increased colony abundance by 32.05% and 113.35% compared to mulching and the control, respectively (Figs 2a, 3a-d, 4a-d). Mulching itself increased colony abundance by 61.57% relative to the control at this depth (Figs 2a, 3a-d, 5a-d, and 7). At a depth of 10 cm, biochar enhanced colony abundance by 42.14% and 42.82% over mulching and the control, respectively (Figs 3c and d, 5c and d, and 7). The treatments with the highest diversity were those treated with biochar, displaying a diversity index of 0.596, while the control exhibited the lowest diversity with an index of 0.422 (Table 1 and Fig 3a-d).

**Fig 6:**
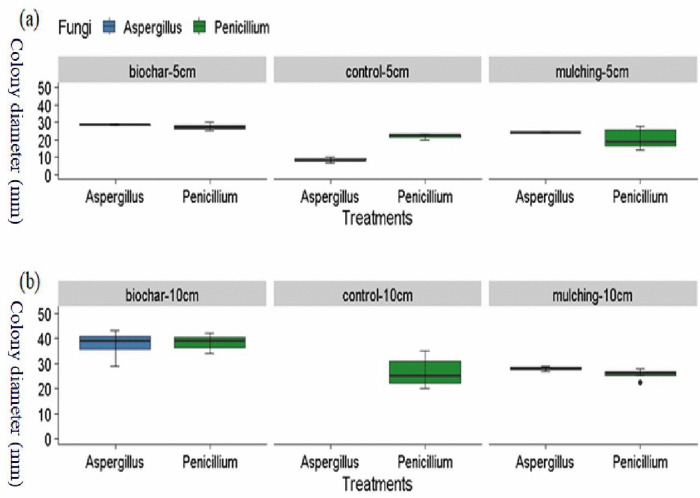
Effect of Biochar and mulching at different soil depth on fungi types at 5 cm (a) and 10 cm soil depth (b). Error bars represent standard errors of the mean (SEM) based on three replicates per treatment.

Biochar and mulching significantly affected (P < 0.05) fungi types colony abundance at different soil depth (Table 2). At 5 cm soil depth Penicillium increased in its fungi colony by 59.45% compared with Aspergillus (Fig 6a). At 10 cm soil depth Penicillium reduced its colony abundance by 21.61% relative to Aspergillus (Fig 6b).

**Table 2:** Diversity of fungi in blocks.

Block	Simpson diversity index
Control	0.422
Biochar	0.596
Mulching	0.536

### 3.2 Earthworm’s appearance frequency

The earthworms appearance frequency was significantly affected (P < 0.05) by biochar and mulching amendment in both seasons (Fig 8a and b). Biochar significantly increased the frequency of earthworms by 84.62% and 37.86% compared with mulching in season one and two, respectively (Fig 8a and b). Biochar significantly increased the frequency of earthworm’s appearance in the plots by 380% and 911% relatively to control, in season one and two, respectively (Fig 8a and b). When compared with control, mulching increased frequency of earthworm’s appearance by 160.00% and 633.33% n season one and two, respectively (Fig 8a and b).

**Fig 7:**
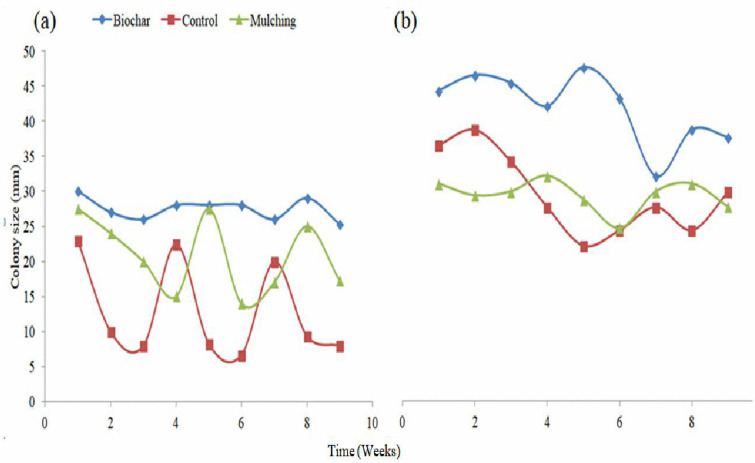
Trend of colony dunamics as affected by biochar and mulching at 5 cm (a) and 10 cm (b) soil depth.

**Fig 8:**
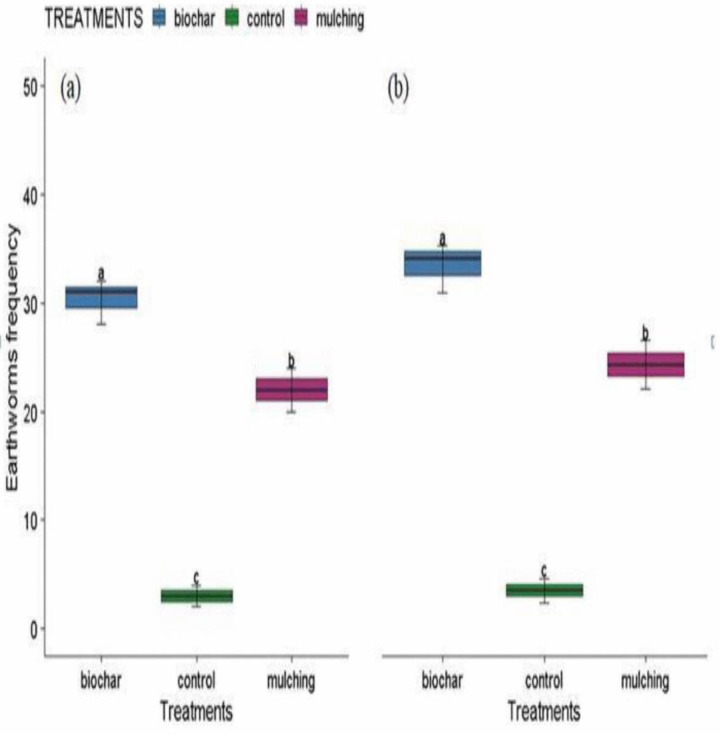
Earthworm's frequency of appearances as affected by biochar and mulching in season one (a) and two (b). Error bars represent standard errors of the mean (SEM) based on three replicatres per treatment.

## 4 Discussion

### 4.1 Effect of biochar and mulching on diversity of fungi

The Simpson diversity index is a key measure of biodiversity, ranging from 0 to 1, where 0 indicates no diversity and 1 represents infinite diversity. In this study, biochar blocks exhibited the highest diversity, with an index of 0.596, followed by mulching at 0.536. The control block showed the lowest diversity at 0.422, consistent with the findings of Winarso et al. [14]. This variation highlights the significant impact of different soil amendments on microbial communities.

Several factors contribute to these differences in microbial diversity. First, biochar provides a stable environment for fungi and bacteria by regulating soil pH, which fosters microbial growth [12]. In contrast, mulches primarily serve as a food source, which may lead to different microbial dynamics. While mulches supply essential nutrients, they do not offer the same level of environmental stability as biochar.

Another important factor is the dynamic nature of biochar. As it decomposes, biochar undergoes changes that allow it to persist longer in the root zone compared to mulches. This extended presence provides microorganisms with more opportunities to interact with biochar, leading to a more diverse microbial community. Conversely, mulch is generally more inert, creating a stable environment that favors certain fungi [15]. This difference in dynamics is crucial for understanding how various amendments affect soil biodiversity.

Organic matter content also plays a key role. Mulch typically contains more organic matter than biochar, affecting the rate of nutrient release into the soil. Microorganisms depend on organic matter as a food source, but the decomposition process can be time-consuming. Microorganisms must first break down this material and consume some nutrients before they become available for plant uptake [16]. This relationship emphasizes the significance of organic matter in microbial activity.

Additionally, biochar creates a more humid environment than mulch, which is particularly beneficial for fungi, as they thrive in moisture-rich conditions. Biochar’s larger surface area enhances soil moisture retention (Fig 7a and b). The study noted that mulching might suppress bacterial growth, providing fungi with a competitive advantage in certain plots. This dynamic interaction reveals the competitive relationships among different microbial species influenced by the type of soil amendment used.

Supporting these findings, other research shows that mulching with wood chips can boost soil fungal diversity by up to 30% [17]. This increase can have positive implications for soil health, as diverse fungal communities are often more resilient and better at nutrient cycling. Furthermore, the study found that mulching not only increased fungal diversity but also the abundance of beneficial fungi compared to the control, enhancing organic matter decomposition and soil structure.

In contrast, research by Francioli et al. [18] indicated that adding biochar to soil increased the biomass of both fungi and bacteria, emphasizing biochar’s potential as a soil amendment to enhance microbial diversity. Overall, these findings highlight the critical role of soil amendments in shaping microbial communities, underscoring the importance of strategic use of such amendments to promote soil health and agricultural productivity.

### 4.2 Effect of biochar and mulching on abundance of *Aspergillus* and *Penicillium*

The study revealed that the fungal colony of *Penicillium* increased at a depth of 5 cm compared to *Aspergillus* (Fig 6a and b). However, at a depth of 10 cm, *Penicillium* experienced a 21.61% reduction in colony abundance relative to *Aspergillus* (Figs 4 and 5). From week one to week three, *Aspergillus* was the most abundant microorganism in all treatments—control, mulching, and biochar—showing particularly high abundance in the 10 cm biochar block. The higher presence of *Aspergillus* at this depth, compared to the mulching and control blocks (Figs 3–5), can be attributed to several factors associated with the properties of biochar.

First, the elevated pH levels of biochar may have created more favorable conditions for *Aspergillus* compared to *Penicillium*. Higher pH can influence microbial growth by enhancing nutrient availability and altering the microbial community structure. Additionally, biochar’s highly porous structure and larger surface area improve soil moisture retention, which likely favors the growth of *Aspergillus* fungi. These findings suggest that both biochar and mulching positively influence microbial community dynamics in the soil, particularly regarding abundance and diversity.

At the conclusion of the mineralization process, biochar was found to retain more nutrients than mulching, attributable to several reasons. First, biochar’s high surface area and porous structure enable it to adsorb and retain nutrients effectively [4]. This creates a nutrient-rich environment that supports the growth of various microorganisms, including *Aspergillus*. Furthermore, the elevated pH of biochar can regulate soil acidity, making the environment more suitable for certain microbial species [19].

Additionally, the physical structure of biochar provides a habitat for microorganisms. This structure offers protected niches and surfaces for colonization and growth, which may particularly benefit *Aspergillus* species [19]. Lastly, biochar can influence soil temperature by absorbing and releasing heat. If *Aspergillus* species are more tolerant of or prefer specific temperature ranges [19], the thermal properties of biochar could further contribute to their higher abundance. Increased temperatures may enhance microbial activity, thereby promoting larger populations and increasing nutrient availability for plants.

Overall, these findings underscore the complex interactions between soil amendments, microbial communities, and nutrient dynamics. The results emphasize the potential benefits of biochar in agricultural systems, particularly in enhancing microbial abundance and diversity. By providing a stable and nutrient-rich environment, biochar can play a critical role in supporting healthy soil ecosystems, ultimately benefiting plant growth and agricultural productivity.

## 5 Conclusion

This study demonstrates that biochar and mulching amendments significantly enhance soil microbial and macro-organism communities in Ethiopian mustard (*Brassica carinata*) cultivation under semi-arid, rainfed conditions. Biochar consistently outperformed mulching and the control in increasing fungal colony abundance, with improvements of 32.05–113.35% at 5 cm depth and 42.14–42.82% at 10 cm depth across two growing seasons. Mulching also provided benefits, boosting fungal abundance by 61.57% over the control at 5 cm depth. Fungal diversity, as measured by the Simpson index, was highest in biochar-treated plots (0.596), followed by mulching (0.536), and lowest in the control (0.422), indicating biochar’s superior role in fostering diverse microbial communities. Earthworm appearance frequency was markedly higher in biochar-amended plots, increasing by 37.86–84.62% compared to mulching and 380–911% relative to the control, underscoring its positive impact on soil macro-organisms. These enhancements can be attributed to biochar’s properties, including improved pH regulation, nutrient retention, porosity, and moisture-holding capacity, which create favorable habitats for fungi (such as *Aspergillus* and *Penicillium*) and earthworms. Notably, *Penicillium* abundance exceeded Aspergillus at 5 cm depth (by 59.45%), while the reverse occurred at 10 cm (with *Penicillium* reduced by 21.61%), highlighting depth-specific responses to amendments. Overall, these amendments promote soil health by supporting organic matter decomposition, nutrient mineralization, and ecosystem resilience, with potential applications beyond Ethiopian mustard to broader cereal cropping systems in acidic, low-fertility soils.
